# Association between intimate partner violence and pregnancy intention: evidence from the Peruvian demographic and health survey

**DOI:** 10.1186/s12905-024-02958-8

**Published:** 2024-02-24

**Authors:** Brenda Caira-Chuquineyra, Daniel Fernandez-Guzman, Andrea G. Cortez-Soto, Diego Urrunaga-Pastor, Guido Bendezu-Quispe, Carlos J. Toro-Huamanchumo

**Affiliations:** 1 Facultad de Medicina, Universidad Nacional de San Agustín de Arequipa, Arequipa, Peru; 2 Carrera de Medicina Humana, Facultad de Ciencias de la Salud, Universidad Científica del Sur, Lima, Peru; 3 Sociedad Científica de Estudiantes de Medicina de Ica (SOCEMI), Universidad Nacional San Luis Gonzaga, Ica, Peru; 4 Unidad de Investigación para la Generación y Síntesis de Evidencias en Salud, Vicerrectorado de Investigación, Universidad San Ignacio de Loyola, Lima, Peru; 5 Escuela de Medicina, Facultad de Ciencias Médicas, Universidad César Vallejo, Trujillo, Peru; 6 OBEMET Center for Obesity and Metabolic Health, Lima, Peru

**Keywords:** Intimate partner violence, Domestic violence, Pregnancy, Epidemiologic factors, Peru

## Abstract

**Background:**

Intimate partner violence (IPV) in Peru represents a significant public health challenge. IPV can influence women’s reproductive and social behaviors, undermining fertility control, and exacerbating unintended pregnancies. Our objective was to assess the association between IPV and pregnancy intention among Peruvian women of reproductive age.

**Methods:**

We conducted a secondary analysis of Peru’s 2020 Demographic and Family Health Survey data. The independent variable in this study was IPV against women, which includes psychological IPV, sexual IPV, and physical IPV. If a respondent experienced any of these three forms of IPV, the IPV variable was labeled as “yes”; if none were present, it was labeled as “no”. The dependent variable was pregnancy intention (no vs. yes). We utilized a generalized linear model (GLM) from the Poisson family with a log link function to assess the relationship between IPV occurrences (total and each IPV type) and pregnancy intention. We report crude and adjusted prevalence ratios (aPR) with 95% confidence intervals (95%CI).

**Results:**

We analyzed data from 8466 women aged 15 to 49. The prevalence of any IPV was 49.6% (psychological IPV: 45.8%; physical IPV: 22.2%; and sexual IPV: 4.3%). Exposure to physical IPV (aPR: 1.05; 95% CI: 1.03–1.07), psychological IPV (aPR: 1.04; 95% CI: 1.02–1.06), and sexual IPV (aPR: 1.09; 95% CI: 1.04–1.13), as well as a history of any IPV (aPR: 1.05; 95% CI: 1.02–1.07), were associated with a higher probability of not intending to become pregnant. This association persisted after adjusting for confounders like age, marital status, educational attainment, education level of the child’s father, place of residence, wealth, ethnicity, and parity.

**Conclusion:**

One in two Peruvian women reported experiencing IPV. An association was observed between IPV exposure and a higher probability of not holding an intention to become pregnant.

**Supplementary Information:**

The online version contains supplementary material available at 10.1186/s12905-024-02958-8.

## Introduction

Intimate partner violence (IPV) is recognized globally as one of the most prevalent forms of violence against women [1]. It encompasses physical, sexual, and psychological harm inflicted by an intimate partner [2]. Globally, an estimated 27% of women aged between 15 and 49 who have been in a relationship report having experienced physical or sexual violence from a partner.

Beyond the immediate physical and psychological repercussions, IPV can profoundly influence a woman’s reproductive and social behaviors, often undermining fertility control, increasing the risk of unwanted pregnancies, coerced abortions, inadequate pregnancy weight gain, preterm deliveries, low birth weight newborns, reduced breastfeeding duration, and heightened neonatal and perinatal mortality [3–5]. The ripple effect of IPV extends further, heightening susceptibilities to cardiovascular, respiratory, neurological, and metabolic disorders, as well as predisposing victims to mood disorders, substance abuse, and suicidal tendencies [6, 7]. Some evidence suggests that IPV survivors might prefer preventing pregnancies, leading to increased contraceptive use [8]. However, in unintended pregnancies, some findings report a reduction in psychological and sexual abuse as the pregnancy progresses, indicating a transient respite around childbirth [9]. However, others have documented persistent or escalated abuse, often driven by paternity disputes [10].

In 2018, South America reported an IPV prevalence of 25%, while estimates for Latin America and the Caribbean varied between 21% and 38% [11]. Specifically, in Peru, 2017 data revealed that 30.6% of women between ages 15 and 49 who were ever married or in cohabitation experienced physical violence, and 6.5% endured sexual violence [12]. Consequently, IPV in Peru is not merely a societal concern but a significant public health challenge, punctuated by alarming rates of abuse and feminicide [13]. This pattern could potentially exacerbate unintended pregnancy rates in a country where abortions, being illegal, frequently occur under dangerous conditions, posing grave health risks to women and sometimes leading to fatalities [14]. While prior research has explored the nexus between IPV and unintended pregnancies among women of reproductive age [15], they often lacked national and only addressed psychological violence—a form of abuse reported as highly prevalent in the region [16]. Therefore, this study aimed to assess the association between IPV experiences and pregnancy intention in the Peruvian context.

## Methods

### Study design

This study is a secondary data analysis from the Peruvian Demographic and Family Health Survey (ENDES) conducted in 2020. The National Institute of Statistics and Informatics (INEI) develops ENDES to capture the socio-demographic and health attributes of the Peruvian population. Data collection involves direct interviews conducted by trained professionals who visit selected households to complete three distinct questionnaires targeting households, women of childbearing age, and heads of households. More information about ENDES methodology is available in the survey technical report [17].

The present manuscript adheres to the STROBE statement guidelines (Strengthening the Reporting of Observational Studies in Epidemiology) [18].

### Population, sample, and sampling

The ENDES is a survey carried out annually with national, urban, and rural representation by the geographical domain (Coast, Sierra, and Jungle) and for the 25 regions of Peru. It employs a two-stage complex probabilistic sampling strategy involving the selection of clusters and then households within them.

**Fig. 1:**
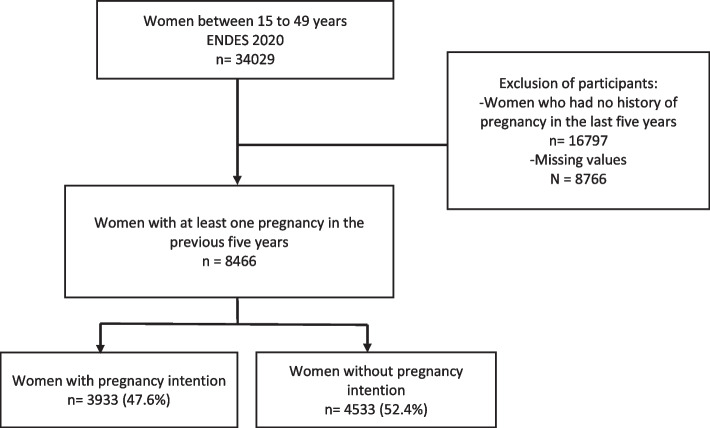
Flowchart for sample selection

Our study targeted women of childbearing age (15–49 years), excluding those who had not registered a pregnancy in the 5 years preceding the survey and those with incomplete information on the variables of interest (Fig. 1). Thus, the data for analysis include women of childbearing age and their pregnancies in the 5 years leading up to ENDES 2019.

### Outcome

The outcome was non-intention to become pregnant. Was determined based on the following question: “When you became pregnant, did you want to get pregnant at that moment, did you want to wait longer, or did you not want to have (more) children?” The response options were: “Yes, at that time”, “Yes, but I wanted to wait”, and “I didn’t want to have (more) children”. For this study, women who selected the first response were categorized as having intended to become pregnant. In contrast, those who chose either of the latter two responses were considered as having not intended to become pregnant.

### Independent variable

The independent variable was IPV. IPV was measured from seven questions related to physical violence, 10 related to psychological or emotional violence, and two about sexual violence committed against the woman by her partner at some point in their relationship (Supplementary material 1). From the data, we derived three binary variables to assess IPV against women: psychological IPV, sexual IPV, and physical IPV, each denoted as “yes” or “no”. A composite IPV variable was then created, encompassing all three types. If a respondent experienced any of the three IPV forms, the composite IPV was labeled as “yes”; if none were present, it was labeled as “no”.

### Other variables

Based on a review of previous studies, we included the following covariates that have been reported to be associated with both variables of interest [8, 19, 20]: mother’s sociodemographic characteristics such as age tertiles (15 to 25 years, 26 to 35, and 36 to 49), current marital status (with a partner and without a partner), education level of the child’s father (initial/preschool/primary, secondary and higher), employment status (works, does not work), geographic region (metropolitan Lima, Costa without Lima, Highlands and Jungle), area of residence (urban and rural), wealth index (first quintile, second quintile, third quintile, fourth quintile, and fifth quintile), ethnicity (mestizo, quechua, negro, and others). Likewise, the father’s education level (initial/preschool/primary, secondary, and higher) was considered. In addition, pregnancy variables such as parity (first child, second child, and third child or more), use of contraceptives prior to pregnancy (no and yes), and the number of prenatal check-ups (PNC) (greater than or equal to six or less than six), as recommended by the Peruvian Ministry of Health [21], were also included. The use of contraceptives before pregnancy and the number of prenatal check-ups were collected for each pregnancy of the surveyed woman, ensuring that the data is not biased by events in other pregnancies.

### Statistical analysis

The 2019 ENDES databases were downloaded and imported into the Stata® v.16.0 program (Stata Corporation, College Station, Texas, USA). The analyses considered the complex sampling and the ENDES weighting factors using the Stata “svy” module. Absolute frequencies and weighted proportions were calculated for the descriptive analysis of categorical variables. We evaluated the relationship between the categorical variables using the chi-square test with the Rao-Scott correction for the bivariate analysis. A generalized linear model (GLM) of the Poisson family with a logarithmic link function was performed to evaluate the association between the presence of IPV (any IPV and for each IPV component) and the intention to become pregnant. In this way, we report the crude prevalence ratios (cPR) and adjusted (aPR) with their respective 95% confidence intervals (95%CI). For the adjusted model, we used an epidemiological approach [22], including the following confounding variables: age, marital status, educational level of both parents, residence, wealth, ethnicity, and parity, whose association has been described in previous studies [8, 19, 20].

Multicollinearity was assessed using the variance inflation factor (VIF) to ensure the reliability of our adjusted regression model. Traditionally, a VIF value greater than 10 indicates substantial multicollinearity between predictor variables. Reassuringly, all variables in our model had VIF values below this threshold. The significance level was set at *p* < 0.05 for all statistical tests.

## Results

We analyzed data from 8466 women aged between 15 and 49 who reported at least one pregnancy within the 5 years preceding ENDES 2019 (Fig. 1).

Most participants were aged between 26 and 35 years (36.7%). A majority were in a relationship (89.6%), had achieved secondary education (46.4%), were employed (63.4%), and resided in metropolitan Lima (33.2%). Nearly 48.9% identified as mestizo, and 34.9% had three or more children. Over 90% had attended six or more PNC (Table 1).

**Table 1:** Characteristics of the study population (*n* = 8466)

Characteristics	n	%^a^	IC95%^a^
Age			
15 to 25 years old	2980	33.0	31.5–34.7
26 to 35 years old	3047	36.7	35.1–38.5
36 to 49 years old	2439	30.3	28.4–31.8
Current marital status			
With partner	7582	89.6	88.5–90.5
Without partner	884	10.4	9.5–11.5
Women’s education level			
Primary or preschool	1648	16.3	15.1–17.6
Secondary	4023	46.4	44.6–48.2
Higher	2795	37.3	35.5–39.1
Education level of the child’s father			
Primary or preschool	1253	12.9	11.8–14.0
Secondary	4360	49.6	47.8–51.4
Higher	2853	37.6	35.7–39.4
Employment condition			
Yes	5313	63.4	61.7–65.0
No	3153	36.6	35.0–38.3
Geographical region			
Lima Metropolitan Area	1145	33.2	31.5–35.0
Rest of coastline	2539	26.0	24.5–27.7
Highlands	2602	24.1	22.4–25.9
Jungle	2180	16.6	15.2–18.0
Residence area			
Urban	5954	77.0	75.7–78.3
Rural	2512	23.0	21.7–24.3
Wealth index			
First quintile	2285	20.2	18.8–21.7
Second quintile	2247	23.6	22.0–25.2
Third quintile	1740	20.6	19.3–21.9
Fourth quintile	1243	17.4	16.0–18.8
Fifth quintile	951	18.2	16.6–19.9
Ethnicity			
Mestizo	3713	48.9	47.2–50.6
Quechua	2279	22.7	21.2–24.2
Negro	893	11.1	10.1–12.2
Others	1581	17.3	16.0–18.8
Parity			
First children	2508	31.4	30.2–32.6
Second children	2771	33.7	32.5–35.0
Third children or more	3187	34.9	33.5–36.3
Contraceptive use			
No	1721	19.4	18.1–20.7
Yes	6745	80.6	79.3–81.9
Number or Prenatal checkups			
> =6	7731	91.1	90.2–92.0
< 6	735	8.9	8.0–9.8
Physical violence			
No	6468	77.8	76.5–79.1
Yes	1998	22.2	20.9–23.5
Psychological violence			
No	4543	54.2	52.4–55.9
Yes	3923	45.8	44.1–47.6
Sexual violence			
No	8090	95.7	95.0–96.2
Yes	376	4.3	3.8–5.0
Intimate partner violence			
No	4180	50.4	48.6–52.2
Yes	4286	49.6	47.8–51.4
Pregnancy intention			
With intention	3933	47.6	45.8–49.3
Without intention	4533	52.4	50.7–54.2

^a^Weighted percentages according to survey complex sampling

The prevalence of psychological, physical, and sexual IPV was 45.8, 22.2, and 4.3%, respectively. A combined IPV prevalence of 49.6% was found, with a higher proportion in women who reported not having a current partner (76.3%; *p* < 0.001), those with less than a higher education level (52.0–54.0%; *p* < 0.001), that their partner or ex-partner had an educational level lower than higher education (53.7–53.9%; *p* < 0.001), those who did not have a labor relationship (52.3%; *p* < 0.001), who belonged to the second and third quintiles of poverty (55.9 and 53.0%; *p* < 0.001). Regarding obstetric characteristics, women with three or more children (55.8%; *p* < 0.001), those who used contraceptives (54.5%; *p* = 0.004), who had less than six PNC (53.0%; *p* < 0.001) and those who did not intend to become pregnant (54.6%, *p* < 0.001), had a higher prevalence of IPV (Table 2).

**Table 2:** Prevalence of intimate partner violence according to the characteristics of the study population (n = 8466)

Characteristics	Intimate partner violence						
	No			Yes			p-value**
	n	%*	IC95%*	n	%*	IC95%*	
Age							
15 to 25 years old	1478	49.0	46.3–51.8	1502	51.0	48.2–53.7	0.394
26 to 35 years old	1542	51.9	48.9–54.9	1505	48.1	45.1–51.1	
36 to 49 years old	1160	50.0	46.5–53.5	1279	50.0	46.5–53.5	
Current marital status							
With partner	3997	53.6	51.7–55.4	3585	46.4	44.6–48.3	**< 0.001**
Without partner	183	23.2	18.8–28.3	701	76.8	71.7–81.2	
Women’s education level							
Primary or preschool	780	48.0	44.5–51.4	868	52.0	48.6–55.5	**< 0.001**
Secondary	1881	46.0	43.5–48.5	2142	54.0	51.5–56.5	
Higher	1519	56.9	53.7–60.1	1276	43.1	39.9–46.3	
Education level of the child’s father							
Primary or preschool	580	46.1	41.9–50.1	673	53.9	49.9–58.1	**< 0.001**
Secondary	2014	46.3	43.9–48.7	2346	53.7	51.3–56.1	
Higher	1586	57.2	54.1–60.4	1267	42.8	39.6–45.9	
Employment condition							
Yes	2484	47.8	45.6–49.9	2829	52.3	50.1–54.4	**< 0.001**
No	1696	54.9	51.9–57.9	1457	45.1	42.1–48.1	
Geographical region							
Lima Metropolitan Area	563	52.2	47.9–56.4	582	47.9	43.6–52.1	0.069
Rest of coastline	1272	49.9	47.0–52.8	1267	50.1	47.2–53.0	
Highlands	1210	46.8	44.3–49.3	1392	53.2	50.7–55.7	
Jungle	1135	52.9	49.7–56.0	1045	47.1	44.0–50.3	
Residence area							
Urban	2920	50.4	48.2–52.5	3034	49.6	47.5–51.8	0.991
Rural	1260	50.4	47.4–53.4	1252	49.6	46.6–52.6	
Wealth index							
First quintile	1140	51.0	47.6–54.4	1145	49.0	45.6–52.4	**< 0.001**
Second quintile	1005	44.1	41.0–47.2	1242	55.9	52.8–59.0	
Third quintile	832	47.0	43.5–50.4	908	53.0	49.6–56.5	
Fourth quintile	650	50.5	46.4–54.6	593	49.5	45.4–53.6	
Fifth quintile	553	61.5	56.1–66.6	398	38.5	33.4–43.9	
Ethnicity							
Mestizo	1964	52.4	49.7–55.1	1749	47.6	44.9–50.3	0.094
Quechua	1007	47.4	44.0–50.8	1272	52.6	49.2–56.0	
Negro	438	49.8	45.2–54.3	455	50.2	45.7–54.8	
Others	771	48.9	44.8–53.0	810	51.1	47.0–55.2	
Parity							
First children	1368	55.2	52.4–58.0	1140	44.8	42.0–47.6	**< 0.001**
Second children	1424	52.3	49.6–54.9	1347	47.7	45.1–50.4	
Third children or more	1388	44.2	41.6–46.9	1799	55.8	53.1–58.4	
Contraceptive use							
Yes	790	45.5	42.1–48.9	931	54.5	51.1–57.9	**0.004**
No	3390	51.5	49.5–53.5	3355	48.5	46.5–50.5	
Number or prenatal checkups							
> =6	3854	50.7	48.9–52.5	3877	49.3	47.5–51.1	**< 0.001**
< 6	326	47.0	41.9–52.2	409	53.0	47.8–58.1	
Pregnancy intention							
With intention	2171	55.8	53.3–58.4	1762	44.2	41.6–46.7	**< 0.001**
Without intention	2009	45.4	42.9–47.9	2524	54.6	52.1–57.1	

*Weighted percentages according to survey complex sampling

**Calculated by Chi2 test of independence with Rao Scott correction for complex sampling. *P*-values < 0.05 are in bold

Over half the participants (52.4%) reported their pregnancy as unintended, with a higher proportion in women aged 15 to 25 years (62.4%; *p* < 0.001), who reported not having a current partner (61.6%; *p* < 0.001), those with educational level below higher education (56.0–56.3%; *p* < 0.001), that their partner or ex-partner had an educational level below higher education (55.0–56.9%; *p* < 0.001), those who resided in rural areas (57.3%; *p* < 0.001), who belonged to the first and second quintiles of poverty (59.4 and 58.0%; *p* < 0.001) and those women with Quechua ethnicity (55.8%; *p* = 0.026). Regarding obstetric characteristics, women who had less than six PNC (62.2%; *p* < 0.001) and those who reported physical IPV (61.5%; *p* < 0.001), psychological IPV (57.6%; *p* < 0.001), sexual IPV (72.2%; *p* < 0.001), and those with any IPV (57.7%; *p* < 0.001), had a higher prevalence of non-intended pregnancy (Table 3).

**Table 3:** Prevalence of non-intended pregnancy according to the characteristics of the study population (*n* = 8466)

Characteristics	Intended pregnancy						
	Intended			Non-intended			p-value**
	n	%*	IC95%*	n	%*	IC95%*	
Age							
15 to 25 years old	1141	37.6	35.0–40.2	1839	62.4	59.8–65.0	**< 0.001**
26 to 35 years old	1529	51.0	48.2–53.7	1518	49.0	46.3–51.8	
36 to 49 years old	1263	54.5	51.0–57.8	1176	45.5	42.2–49.0	
Current marital status							
With partner	3601	48.7	46.8–50.6	3981	51.3	49.4–53.2	**< 0.001**
Without partner	332	38.4	33.9–43.1	552	61.6	56.9–66.1	
Women’s education level							
Primary or preschool	715	44.0	40.5–47.5	933	56.0	52.5–59.5	**< 0.001**
Secondary	1769	43.7	41.4–46.0	2254	56.3	54.0–58.6	
Higher	1449	54.1	50.9–57.2	1346	45.9	42.8–49.1	
Education level of the child’s father							
Primary or preschool	537	43.1	38.9–47.4	716	56.9	52.6–61.1	**< 0.001**
Secondary	1930	45.0	42.8–47.2	2430	55.0	52.8–57.2	
Higher	1466	52.6	49.4–55.7	1387	47.4	44.3–50.6	
Employment condition							
Yes	2468	47.4	45.1–49.6	2845	52.6	50.4–54.9	0.711
No	1465	48.0	45.4–50.7	1688	52.0	49.3–54.6	
Geographical region							
Lima Metropolitan Area	545	50.3	46.4–54.3	600	49.7	45.7–53.6	0.073
Rest of coastline	1213	47.4	44.4–50.3	1326	52.6	49.7–55.6	
Highlands	1202	46.6	44.0–49.3	1400	53.4	50.7–56.0	
Jungle	973	43.9	40.6–47.2	1207	56.1	52.8–59.4	
Residence area							
Urban	2876	49.1	47.0–51.2	3078	50.9	48.8–53.0	**< 0.001**
Rural	1057	42.7	39.7–45.6	1455	57.3	54.4–60.3	
Wealth index							
First quintile	940	40.6	37.4–44.0	1345	59.4	56.0–62.6	**< 0.001**
Second quintile	961	42.0	39.0–45.1	1286	58.0	54.9–61.0	
Third quintile	858	49.8	46.5–53.2	882	50.2	46.8–53.5	
Fourth quintile	639	48.9	44.8–53.0	604	51.1	47.0–55.2	
Fifth quintile	535	58.9	53.4–64.1	416	41.1	35.9–46.6	
Ethnicity							
Mestizo	1807	49.9	47.3–52.5	1906	50.1	47.5–52.7	**0.026**
Quechua	996	44.2	41.0–47.3	1283	55.8	52.7–59.0	
Negro	422	47.6	43.2–52.0	471	52.4	48.0–56.8	
Others	708	45.6	41.8–49.5	873	54.4	50.5–58.2	
Parity							
First children	1228	50.8	48.0–53.6	1280	49.2	46.4–52.0	**< 0.001**
Second children	1515	55.3	52.8–57.8	1256	44.7	42.2–47.2	
Third children or more	1190	37.2	34.6–39.8	1997	62.8	60.2–65.4	
Contraceptive use							
No	804	47.4	44.2–51.0	917	52.6	49.0–55.8	0.896
Yes	3129	47.6	45.6–49.6	3616	52.4	50.4–54.4	
Number or ANC visits							
> =6 ANC	3657	48.6	46.8–50.3	4074	51.5	49.7–53.2	**< 0.001**
< 6 ANC	276	37.8	33.0–42.8	459	62.2	57.2–67.0	
Physical violence							
No	3182	50.2	48.2–52.2	3286	49.8	47.8–51.8	**< 0.001**
Yes	751	38.5	35.4–41.6	1247	61.5	58.4–64.6	
Psychological violence							
No	2325	52.0	49.4–54.6	2218	48.0	45.4–50.6	**< 0.001**
Yes	1608	42.4	40.1–44.8	2315	57.6	55.2–59.9	
Sexual violence							
No	3825	48.5	46.7–50.3	4265	51.5	49.7–53.3	**< 0.001**
Yes	108	27.8	21.9–34.5	268	72.2	65.5–78.1	
Intimate partner violence							
No	2171	52.8	50.1–55.4	2009	47.2	44.6–49.9	**< 0.001**
Yes	1762	42.4	40.1–44.6	2524	57.7	55.4–59.9	

*Weighted percentages according to survey complex sampling

**Calculated by Chi2 test of independence with Rao Scott correction for complex sampling. *P*-values < 0.05 are in bold

In the adjusted regression model, after adjusting for potential confounders, having experienced physical IPV (aPR: 1.05; 95% CI: 1.03–1.07), psychological IPV (aPR: 1.04; 95% CI: 1.02–1.06), and sexual IPV (aPR: 1.09; 95% CI: 1.04–1.13), as well as a history of any IPV (aPR: 1.05; 95% CI: 1.02–1.07), were associated with a higher probability of not intending to become pregnant (Table 4).

**Table 4:** Association between intimate partner violence and non-intended pregnancy, Peru, 2020

Characteristics	Crude Model			Adjusted Model^a^		
	cPR	95%CI	*p*-value	aPR	95%CI	*p*-value
Intimate partner violence						
No	Ref.			Ref.		
Yes	1.07	1.05–1.10	**< 0.001**	1.05	1.02–1.07	**< 0.001**
Physical violence						
No	Ref.			Ref.		
Yes	1.08	1.05–1.10	**< 0.001**	1.05	1.03–1.07	**< 0.001**
Psychological violence						
No	Ref.			Ref.		
Yes	1.06	1.04–1.09	**< 0.001**	1.04	1.02–1.06	**0.001**
Sexual violence						
No	Ref.			Ref.		
Yes	1.14	1.09–1.18	**< 0.001**	1.09	1.04–1.13	**< 0.001**

Odds ratios and confidence intervals were calculated considering the survey complex sampling. *P*-values < 0.05 are in bold

*cPR* crude Prevalence Ratio, *aPR* adjusted Prevalence Ratio

^a^Model adjusted by age, marital status, educational level, partner’s educational level, residence, wealth, ethnicity, and parity

## Discussion

We sought to assess the association between intimate partner violence and pregnancy intention among childbearing-age women in Peru. Half of the participants had experienced IPV, with psychological IPV being the most prevalent. Additionally, a relationship was found between IPV and pregnancy intention; women who experienced any form of IPV were less likely to intend for pregnancy.

The prevalence of specific types of IPV in this study contrasts with prior results. For instance, a systematic review from 2017 revealed that Peru had a prevalence of 30.6% for physical IPV and 6.5% for sexual IPV [12], both higher than our current findings. An analysis by INEI of the ENDES data spanning 2009 to 2018 indicated that psychological (73.0 to 58.9%), physical (38.2 to 30.7%), and sexual (8.8 to 6.8%) decreased in this period (any form of IPV: 76.9 to 63.2%) [23]. Tiravanti-Delgado et al.’s 2019 study using the ENDES reported a general IPV prevalence of 57.7%, psychological at 52.8%, physical at 29.5%, and sexual at 7.1%, higher than our results for the same year [24]. Their study had a larger sample of 21,518 women of reproductive age compared to our 8466, which might account for the discrepancies. The diminishing IPV prevalence over the years in Peru is corroborated by our results. This decrease in the IPV may be the cause of a greater awareness of the equal rights of women and the public policies against violence against women by the Peruvian government, such as the National Agreement adopted in 2002 in its policies 7 and 16, the Strategic Plan for National Development in its “Bicentennial Plan: Peru towards 2021” approved by Supreme Decree 054–2011-PCM in its axis 1 and 2, and in the National Policies approved by Supreme Decree 056–2018-PCM in its priority guideline No. 4.6 [25, 26].

Half of the women we evaluated had no intention of becoming pregnant after experiencing IPV, a prevalence higher in rural areas. This is a decrease from a 2012 study, where 62.3% of urban and 74.1% of rural women became unintentionally pregnant [27]. This decreasing trend in unintended pregnancies could be due to enhanced informational campaigns, better contraceptive access, and evolving reproductive aspirations in Peru.

Women exposed to any form of IPV (psychological, physical, or sexual) have a higher prevalence of unwanted pregnancies. This pattern aligns with findings from various countries: Ethiopia [28], Bangladesh [27, 29], Brazil [27, 30], Japan, Nabidia, Samoa, Serbia and Montenegro, Thailand, Tanzania [27], Spain [31], and previous studies in Peru [27]. A likely reason is the dominance exerted by abusive partners, curtailing women’s fertility control and reinforcing submissive dynamics in sexual relations, subsequently leading to unwanted pregnancies [19]. The relationship between unwanted pregnancies and contraceptive use in this context is multifaceted. Some women, fearing further abuse during pregnancy or succumbing to pressures from partners or in-laws, avoid contraceptives altogether [32]. On the other hand, having suffered from violence promotes the use of contraceptive methods to avoid getting pregnant without the partner knowing, the use of emergency contraception in the event of a forced attempt to have sexual intercourse, or opting for abortion in the case of get pregnant without their consent [33]. This clandestine use of abortion is intrinsically linked to IPV: women exposed to violence often report higher abortion rates than those who are not [19]. However, in Peru, these statistics might not reflect reality. Since only therapeutic abortion is legal, many IPV-induced abortions could go unreported in medical records.

The evident link between IPV and unintended pregnancies underscores the need for robust prevention programs against violence directed at women, safeguarding their well-being and reproductive autonomy. Identifying signs of physical, sexual, or psychological violence is critical. Often, women in dependent relationships or those with low self-esteem might downplay or overlook the true extent of the abuse they face [34]. Equally vital is promoting sex education, family planning, and accurate information about contraceptive methods. In Peru, misconceptions persist about different contraceptive options, their usage, and potential side effects. Additionally, local support programs should be prioritized. These initiatives should provide information on IPV and offer guidance on reporting violence and seeking help. Creating an environment that fosters a sense of belonging, acceptance, and destigmatization for victims is essential. Such supportive environments have been shown to correlate with reduced rates of unintended pregnancies among abused women [35].

This study has some limitations. First, this study is a secondary analysis of a public database, so the accuracy of all the data analyzed cannot be guaranteed. However, the ENDES is a widely used survey with quality controls that allow the study of the health status of the Peruvian population, being used by researchers and authorities to study health problems in the Peruvian context. Second, there may be a social desirability bias on the part of the respondents due to how sensitive it may be to provide information regarding violence issues, which could underestimate the presence of violence in the population studied. Third, due to the survey’s design (cross-sectional study), it is impossible to establish a causal relationship between the variables of interest.

## Conclusion

Half of the Peruvian women in our study experienced IPV, with psychological IPV being the most prevalent. Exposure to IPV increases the likelihood of not intending to get pregnant. These findings underscore the urgency of reinforcing Peru’s ongoing preventive measures against IPV and maternal health strategies. Comprehensive sexual education and systematic IPV monitoring are essential to address this concern. Furthermore, it is crucial to inculcate respect and gender equality from an early age.

### Supplementary Information

**Additional file 1: Supplementary Material 1.** Questions Related to intimate partner violence in ENDES (In parentheses the English translation).

## Data Availability

This study performed a secondary analysis of a publicly available access database from the INEI (https://proyectos.inei.gob.pe/microdatos/).

## Abbreviations

IPV: Intimate partner violence
ENDES: Demographic and family health survey
INEI: National institute of statistics and informatics
STROBE: Strengthening the reporting of observational studies in epidemiology
PNC: Prenatal care
GLM: Generalized linear model
Poisson: Poisson distribution
VIF: Variance inflation factor
cPR: Crude prevalence ratio
aPR: Adjusted prevalence ratio
95% CI: 95% confidence interval.
