# Supplementary material for: Association between intimate partner violence and pregnancy intention: evidence from the Peruvian demographic and health survey
Source: BMC Womens Health. 2024 Feb 24;24:140. doi: 10.1186/s12905-024-02958-8 (PMC10893598; doi:10.1186/s12905-024-02958-8)
Supplement: Supplementary file 1 — Additional file 1: Supplementary Material 1. Questions Related to intimate partner violence in ENDES (In parentheses the English translation). [file 12905_2024_2958_MOESM1_ESM.docx]

**Supplementary Material 1. Questions Related to intimate partner violence in ENDES (In parentheses the English translation).**

| **Preguntas Relacionadas a Violencia psicológica o emocional** *(Questions Related to Psychological or Emotional Violence)* | **Respuestas** *(Answers)* |
| --- | --- |
| 1.      ¿Su esposo (compañero) se pone (ponía) celoso o molesto si usted conversa (conversaba) con otro hombre?  *(Does your husband (partner) get (get) jealous or upset if you talk (talked) with another man?)* | a. No *(No)* b. Sí *(Yes)* c. No sé *(Don’t know)* |
| 2.      ¿El la acusa (acusaba) frecuentemente de ser infiel?  *(Does he frequently accuse (accuse) you of being unfaithful?)* |  |
| 3.      ¿El le impide (impedia) que visite o la visiten sus amistades?  *(Does he (prevents) you from visiting or visiting you friends?)* |  |
| 4.      ¿El trata (trataba) de limitar las visitas/contactos a su familia?  *(Does he try to limit visits/contacts of your family?)* |  |
| 5.      ¿El insiste (insistía) siempre en saber todos los lugares donde usted va (iba)?  *(Does he always insist (insisted) on knowing all the places where you go (went)?)* |  |
| 6.      ¿El desconfía (desconfiaba) de usted con el dinero?  *(Does he mistrust (mistrust) you with money?)* |  |
| 7.      ¿Le ha dicho o le ha hecho cosas para humillarla delante de los demás?  *(Has he said or done things to put you down in front of others?)* | a. No *(No)* b. Sí *(Yes)* |
| 8.      ¿La ha amenazado con hacerle daño a usted o a alguien cercano a usted?  *(Threatened to hurt you or someone close to you?)* |  |
| 1. ¿Le insulta o hace sentir mal?   *(Does he insult you or make you feel bad?)* |  |
| 1. ¿La ha amenazado con irse de la casa, quitarle a las hijas e hijos o la ayuda económica?   *(Has he threatened to leave the house, take away your daughters and sons, or financial aid?)* |  |
| **Preguntas relacionadas a violencia física** *(Questions related to physical violence)* | **Respuestas** *(Answers)* |
| 1.     ¿La empujó, sacudió o le tiró algo?  *(Pushed, shook, or threw anything at you?)* | a. No *(No)* b. Sí *(Yes)* |
| 2.     ¿La abofeteó o le retorció el brazo?  *(Did he slap or twist your arm?)* |  |
| 3.     ¿La golpeó con el puño o con algo que pudo hacerle daño?  *(Did he hit you with his fist or with something that could hurt you?)* |  |
| 4.     ¿La ha pateado o arrastrado?  *(Has he kicked or dragged you?)* |  |
| 5.     ¿Trató de estrangularla o quemarla?  *(Did he try to strangle or burn you?)* |  |
| 6.     ¿La atacó/agredió con un cuchillo, pistola u otro tipo de arma?  *(Did he attack/assault you with a knife, gun, or other type of weapon?)* |  |
| 7.     ¿La amenazó con un cuchillo, pistola u otro tipo de arma?  *(Threatened you with a knife, gun, or other type of weapon?)* |  |
| **Preguntas relacionadas a violencia sexual** *(Questions related to sexual violence)* | **Respuestas** *(Answers)* |
| 1.     ¿Ha utilizado la fuerza física para obligarla a tener relaciones sexuales aunque usted no quería?  *(Has he used physical force to make you have sex when you didn't want to?)* | a. No *(No)* b. Sí *(Yes)* |
| 2.      ¿La obligó a realizar actos sexuales que usted no aprueba?  *(Forced you to perform sexual acts that you do not approve of?)* |  |
